# Blood glutamate scavenging as a novel glutamate-based therapeutic approach for post-traumatic brain injury anxiety and social impairment

**DOI:** 10.1038/s41398-023-02329-1

**Published:** 2023-02-04

**Authors:** Dmitry Frank, Benjamin F. Gruenbaum, Ilan Shelef, Vladislav Zvenigorodsky, Olena Severynovska, Ilya Fleidervish, Boris Knyazer, Amit Frenkel, Alexander Zlotnik, Ora Kofman, Matthew Boyko

**Affiliations:** 1 Department of Anesthesiology and Critical Care, Soroka University Medical Center, Ben-Gurion of the Negev, Beer-Sheva, Israel; 2 Department of Anesthesiology and Perioperative Medicine, Mayo Clinic, Jacksonville, FL USA; 3 Department of Radiology, Soroka University Medical Center, Ben-Gurion of the Negev, Beer-Sheva, Israel; 4 Department of Biochemistry and Physiology of the Faculty of Biology and Ecology, Oles Gonchar of the Dnipro National University, Dnipro, Ukraine; 5 Department of Physiology and Cell Biology, Faculty of Health Sciences and Zlotowski Center for Neuroscience, Ben–Gurion University of the Negev, Beer-Sheva, 84105 Israel; 6 Department of Ophthalmology, Soroka University Medical Center and the Faculty of Health Sciences, Ben-Gurion University of the Negev, Beer-Sheva, Israel; 7 Psychology Department, Zlotowski Center for Neuroscience, Ben-Gurion University of the Negev, POB 653, Beer-Sheva, 84105 Israel

**Keywords:** Molecular neuroscience, Physiology

## Abstract

Traumatic brain injury (TBI) is a serious condition that is associated with an increased risk of severe, long-term psychiatric consequences. Drugs that target the glutamatergic system have proven successful in treating both TBI and many of its psychiatric sequelae. Blood glutamate scavengers (BGS) cause a decrease in blood glutamate levels, leading to a reduction in glutamate’s concentration gradient from the brain to the blood and decreased levels of brain glutamate. This study evaluated the BGS pyruvate as a treatment for TBI-related neuropsychiatric conditions in a rat model. 213 rats were divided into four groups in a 2 × 2 design: Sham or TBI rats treated with pyruvate or control treatment. Magnetic resonance imaging, neurological status, brain glutamate and blood glutamate levels were assessed following the injury. Four weeks after the start of treatment, all rats underwent behavioral tests to assess anxious behavior and social impairment (aggressive and hierarchical behavior). Rats responded positively to pyruvate in several tasks, lowering brain glutamate levels and reducing anxiety and depression, as well as modulating TBI-related changes in social behavior. Glutamate scavenging with pyruvate may be an effective therapeutic option for post-TBI behavioral changes by reducing associated elevations in brain glutamate levels.

## Introduction

Traumatic brain injury (TBI) is a serious condition that requires significant economic resources for treatment, both for the individual patient and for the healthcare system. Patients with TBI need to be treated for the proximal effects of the trauma and for long-term effects, which can include life-long disability, as experienced by more than five million people in the United States [1].

TBI is also associated with an increase in severe, long-term psychiatric disorders. Among those with moderate to severe TBI, nearly half (49%) have shown indications of psychiatric illness within the first year after TBI, compared with 34% of those with mild TBI and 18% in the general population without TBI [2]. These psychiatric illnesses commonly include major depression, generalized anxiety disorder, post-traumatic stress disorder, social withdrawal, apathy, or aggression [3, 4]. TBI survivors can suffer these disorders for decades following the brain injury [5, 6], causing major difficulties to lifestyle and health through disruptions to rehabilitation efforts [7].

Anxiety disorders, which occur in ~18% of the adult population, are often co-morbid with other psychiatric disorders and are associated with an elevated risk of suicide, and increased economic and social burden [8]. Treatment involves pharmaceutical or behavioral therapies, such as cognitive behavioral therapy, which fails or leads to incomplete relief in 46.4% of patients [8]. First-line pharmacological treatment for DSM-5 anxiety disorders primarily consists of antidepressants that modulate serotonin neurotransmission such as selective serotonin reuptake inhibitors (SSRIs) and serotonin norepinephrine reuptake inhibitors (SNRIs) [8]. Serotonergic antidepressants, though, have a number of adverse effects, such as nausea, diarrhea, diaphoresis, headaches, tremor, asthenia, insomnia, and somnolence [9]. Abruptly stopping antidepressant treatment can also result in a withdrawal syndrome characterized by increased anxiety symptoms, panic attacks, dizziness, and nausea [10].

It is critical to find alternative therapies to regulate symptoms of psychiatric illnesses. Drugs targeting the regulation of the glutamate system have shown potential to treat anxiety disorders [11]. Glutamate, due to its role as a neurostimulator, has a vital impact on cognition, learning, and mood, areas that rely on neuroplasticity to assist with adapting to environmental stressors [12, 13]. In particular, chronic stress can result in dysregulation of the glutamate system and reduced neuroplasticity. This may be due to activation of the microglial cells caused by stress, resulting in neuroinflammation and having an effect on both intracellular and extracellular signaling pathways [14].

The glutamatergic system appears to play a role in many neurodegenerative conditions [15], such as TBI [16] and stroke [17], and the relationship between glutamate and behavioral and psychiatric disorders has also been well-established [18]. The metabolic pathway of glutamate has been associated with depression, obsessive-compulsive disorder (OCD) and post-traumatic stress disorder [19, 20], which often co-occur with anxiety disorders. Modified glutamate levels have been observed in post-mortem studies on depressed patients, located in plasma, cerebrospinal fluid (CSF), and brain tissue [21]. Possible benefits of glutamate in the treatment of depression following stroke has been observed in animal studies [22] and supported by genetic studies that found links between genes associated with glutamatergic transmission and depression [23]. Similarly, sufferers of OCD have been observed with abnormal levels of glutamate in CSF [20]. Some studies of elevated glutamate levels for OCD patients and patients with depression, however, have been inconsistent [24].

The mechanisms behind increases in glutamate following TBI can result in a number of adverse effects. As the most abundant free amino acid in the brain [25], glutamate has a concentration in plasma of 50–100 µM/L, a concentration of about 12,000 µM/g in the whole brain [26], and a concentration of only 0.5–2 µM/L in extracellular fluids [27]. Increased brain glutamate is associated with neuronal death, destruction of the blood brain barrier (BBB), inflammation, and stress [16]. Immediately following TBI, cerebral glutamate increases its concentration, then decreases, but does not return to baseline levels. The increased levels can remain for many months or years, mainly due to breakdown of the BBB [16]. The regulation of glutamate is an important approach in controlling the effects of brain damage.

Mitigating the toxic effects of excessive glutamate has been a major goal in research studies, aimed at minimizing damage and improving recovery after TBI. Neurological motor symptoms of TBI were attenuated by limiting excess glutamate with dextorphan [28], n-methyl-D-aspartate (NMDA) antagonists [29], stimulation of Na + -dependent excitatory amino acid transporters [30], or antibiotics and other drugs that block calcium channels or glutamate release [31]. Clinical success has been limited and occasionally marked by adverse side effects [32]. Other studies have found that direct or indirect stimulation of NMDA receptors mitigated the severity of neurological signs and deficits in hippocampal-based memory in adult rats [33] and in rat pups [34] following TBI. There are no studies in the literature yet that have used the blocking or augmentation methods to test their results on the psychopathological symptoms of anxiety and aggression following TBI.

Excess brain glutamate can also be mitigated by manipulating the brain-blood glutamate equilibrium and inducing excess glutamate from the brain’s interstitial fluid to flow into the body’s circulatory system [15]. Glutamate is inactivated by blood glutamate scavengers (BGS), such as oxaloacetate and pyruvate, that convert the amino acid into 2-ketoglutarate, causing a decrease in glutamate blood levels and subsequently a lower brain-blood concentration gradient and decreased levels of brain glutamate at 24 and 48 h after traumatic brain injury [35]. This relationship makes this method especially relevant in conditions that penetrate the BBB, such as TBI, stroke, and chronic stress [15]. However, the ability of this method to mitigate emotional and cognitive deficits has not been investigated.

In addition to its success in limiting excess glutamate and the neurological and immunological signs of traumatic injury to the brain and spinal cord [36], BGS effectively reduces the neurological and behavioral symptoms of subarachnoid hemorrhage [37] and middle cerebral artery occlusion [18]. BGS preserves the physiological effects of glutamate in regulating the metabolic and electrolyte balance, maintaining neuronal integrity, and improving neuro-repair after brain injury. The principal aim of this study is to investigate effect of BGS treatment on the recovery of anxiety and emotional functions following TBI in rats.

## Materials and methods

### Animals

The experiments were conducted in accordance with the recommendation of the Declarations of Helsinki and Tokyo and to the Guidelines for the use of Experimental Animals of the European Community. The experiments were approved by the Animal Care Committee of Ben-Gurion University of the Negev, Israel. A total of 273 Sprague-Dawley rats (Envigo Laboratories, Israel) weighing between 280 g and 320 g were used in this experiment (Table 1). Rats had access to Purina Chow and water ad libitum. The temperature in the room was maintained at 22 °C, with a 12 h light–dark cycle, and all tests were conducted in the dark phase between 8 a.m.–4 p.m.

**Table 1:** The total number of rats in each of the experimental groups (excluding 6 male and 7 female rats that died, as discussed in the Results section).

Study groups	Experimental procedures		Number of rats	
	MRI, CSF, and blood collection	Neuro-behavioral tests	Female	Male
Sham-operated controls given pyruvate	10 f	15 f	25	25
	10 m	15 m		
Sham-operated controls given placebo	10 f	15 f	25	25
	10 m	15 m		
Post-TBI rats given placebo	10 f	15 f	25	25
	10 m	15 m		
Post-TBI rats treated with pyruvate	10 f	15 f	25	25
	10 m	15 m		
Naïve rats for resident-intruder paradigm		30 f	30	30
		30 m		
The total number of rats			130	130

### Drugs and doses

Pyruvate (Sigma Israel Chemicals, Rehovot, Israel) was kept at a temperature of 2^o^–4 °C prior to use. Immediately before administration, it was dissolved in drinking water, with a new solution prepared every 12 h. Doses of 180 mg/kg/day were administered to rats in the experimental groups divided into two daily doses of 90 mg/kg for 28 days. This dose was established from data that showed through magnetic resonance spectroscopy that a dose of 180 mg/kg/day was optimal for reducing blood and brain glutamate by about 25–35% [22, 38]. The placebo groups received an equal dose of water with no pyruvate.

### Experimental design

**Fig. 1:**
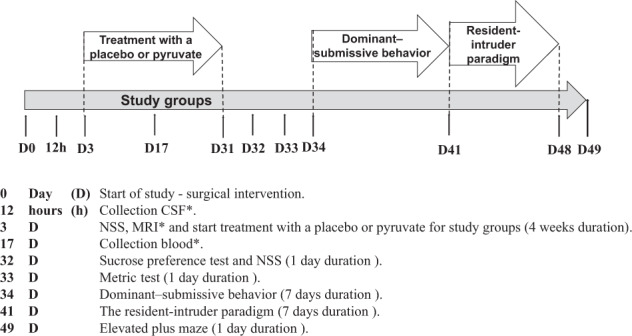
Experimental timeline. *Rats subjected to behavioral tests did not undergo MRI, CSF collection, or blood collection. CSF Cerebrospinal fluid; MRI Magnetic resonance imaging; NSS Neurological severity score.

The study was run using a 2 × 3 design. The dependent variables were TBI (113 rats) sham (100 rats), treatment (control/pyruvate) and sex (male/female), with 15 rats/group. To test aggression, 60 additional naïve rats (30 females, 30 males) were used in the resident-intruder test (Table 1). Female rats were housed with males to enhance aggression [39] and intruder males were introduced as stimulus rats to provoke aggression. Rats with neurological deficits after 4 weeks were excluded from the study so that the behavioral results would not be confounded by motor deficits. Magnetic resonance imaging and neurological status were assessed 72 h after the intervention. Brain glutamate was measured 12 h after the intervention, and blood glutamate level was assessed 14 days after the start of treatment. Four weeks after the start of treatment, all rats from each experimental group underwent a series of behavioral tests (Fig. 1).

### Induction of TBI

TBI was performed, as previously described [40–43]. Rats received inhaled isoflurane as anesthetic with 5% for induction and 1.5–2.5% for maintenance, with equal amounts of medical air and oxygen. Prior to incision, the scalp was infiltrated with 0.5% bupivacaine. It was then perforated and reflected laterally with the left temporal muscle, while the underlying periosteum was dissected to expose the skull. Craniotomy was performed at 5-mm using a trephine (Roboz Surgical Instrument Co., Gaithersburg) connected to the drill bit of an electrical drill (Stoelting Wood Dale, IL). The center of the craniotomy was situated 4 mm lateral and 4 mm posterior to bregma. A Luer 3-way stopcock was fixed and additionally held in place by cyanoacrylate adhesive and dental acrylic. The injury was then induced by a pressure pulse of 2.2 atmospheres [40, 41] with TBI effected by a fluid-percussion device over 21-23 msec through the 3-way stopcock. The fluid pulse from the piston plunger, through the pendulum’s mechanism, was enacted via continuous saline fluid into the dura to allow for efficient transmission of the pressure pulse. Rats in the sham-operated control groups underwent the same procedure but without the administration of the fluid pulse.

Rats were monitored by a pulse-oximeter during the surgery to ensure accurate and uninterrupted measurements of heart rate and blood oxygen levels. After TBI induction, the incision was sutured, and the rats were allowed to recover from anesthesia.

### Determination of blood glutamate

Blood (200 μl) was collected from the tail vein to measure blood glutamate levels via a 24-gauge Neoflon (Becton Dickinson Helsingborg, Sweden) catheter. After the blood sample was collected, the catheter was removed from the vein [37]. Blood preparation and analysis were performed by the fluorometric method [38, 44].

### Determination of CSF glutamate

CSF (110 μl) was mixed with perchloric acid (25 μl) of 0.3 M, and then centrifuged at 10,000 × g for 10 min at 4 °C. The pellet was discarded and the supernatant was collected, adjusted to pH 7.2 with 12.5 μl of 2 M K2CO3 and stored at -80 °C for analysis at a later time [37]. Analysis was performed by the same fluorometric method used for blood samples [44].

### Neurological severity score (NSS)

Two blinded observers calculated NSS, as previously described [43, 45–48]. Points were assigned for motor function and behavioral changes for an overall score between 0, which represented an intact neurological state, and 25, for the highest neurological impairment. The following criteria were evaluated: the ability to exit a circle (3-point), gait on a wide surface (3-point), gait on a narrow surface (4-point), effort to remain on a narrow surface (2-point), reflexes (5-point), seeking behavior (2-point), beam walking (3-point), and beam balance (3-point).

### MRI study

MRI was utilized for T2 and diffusion-weighted imaging at 72 h following TBI, as described previously [43]. A 3 T MRI was used (Ingenia, Philips Medical Systems, Best, The Netherlands) using an eight-channel receive-only coil. The Intellispace Portal workstation (V5.0.0.20030 Philips Medical Systems, Best, The Netherland) performed the post-processing of the permeability and perfusion studies.

### MRI analysis

Image analysis was performed by an expert blinded to the group assignments. The calculation of the lesion volume with the correction for tissue swelling was done using the following formula:[49] corrected lesion volume = (lesion volume × contralateral hemisphere size)/(ipsilateral hemisphere size).

The calculation of brain edema was done by comparing the contralateral and ipsilateral hemispheres, and performed using the following formula:[46] brain edema = (volume of the ipsilateral hemisphere-volume of the contralateral hemisphere)/(volume of the contralateral hemisphere).

### Sucrose preference test

The sucrose preference test was performed as described previously [50] to evaluate anhedonia, an established rodent model of depression. Two bottles of a 1%(w/v) sucrose solution were placed in each rat’s cage. One of the bottles was replaced with water for 24 h so that the rat could adjust to having one bottle of water and one bottle of sucrose. After this habituation, the rats were deprived of food and water for 12 h and then given 4-h access to two bottles, one with 100 ml of sucrose solution (1% w/v) and the other with 100 ml of water. The volume (ml) consumed from each bottle was recorded and sucrose preference as sucrose consumption (ml)/(sucrose consumption (ml)+water consumption(ml)) × 100% [50].

### Elevated plus maze task

The plus maze was situated in a dark room and consisted of two open and two closed arms (each measuring 16 × 46 cm). It was constructed from black plastic and positioned 100 cm above the floor. The closed arms, facing one another, were each enclosed by a 40 cm high wall. 10% ethanol was used to clean the maze prior to each animal’s entry. In random order, each rat was placed in the center of the plus maze facing one of the open arms, and the rat’s behavior was videotaped(Logitech_HD_Pro_Webcam_C920) for 5 min and analyzed offline for entries and time spent in the open arms using Ethovision XT software (Noldus_Wageningen_Netherlands) [51].

### Dominant-submissive behavior

Seven days before testing, the rats were randomly divided into cages containing 1 sham-operated and 1 TBI rat. On each of the 2 days prior to the test, the rats were habituated to the device for 15 min. The apparatus consisted of two transparent Plexiglas boxes (30 cm × 20 cm × 20 cm) connected by a narrow passage (15 cm × 15 cm × 60 cm). A feeder containing sweetened milk was placed in the middle of the passage [52, 53]. Only one rat was able to fit in the feeder area at a time. During the testing period, the rats only received food in the apparatus. The pair of rats were each placed equidistance to the feeder, and their behavior was filmed for 5 min. The time that each rat spent at the feeder and the first rat to arrive at the feeder were recorded from the video on the 5th day.

### Resident-intruder paradigm

The resident-intruder paradigm, a standardized test for aggression and social stress, was performed and calculated by two blinded observers as previously described [52, 54]. TBI and sham-operated male rats were housed with naïve companion females that were not siblings in a polycarbonate cage with a surface area of half a square meter for 7 days prior to testing with free access to food and water. Bedding was not changed during that week or during testing.

One hour before the test, the female companions were removed from the cage. An hour later, an unfamiliar naïve male was placed in the cage with the original male. The interactions of the two rats were video recorded for 10 min, including the duration and frequency of behavioral parameters. Rats were rated on the following behaviors: Offensive behavior (rearing; attack latency, the time between the introduction of the intruder and the first clinch attack; social exploration; ano-genital sniffing; lateral threat; upright posture; clinch attack; keep down; move towards; chase), defensive behavior (non-social explore, submission latency, submissive posture, flight, defensive upright posture, freeze, move away) and rest or inactivity [54]. Following testing, the male intruder was removed from the cage and the original male resident was reunited with its original companion female.

### Statistical analysis

Statistical analysis was performed with the SPSS-22 and Statistica-12 (StatSoft) package. A Kolmogorov–Smirnov test was used to decide the appropriate test for the comparisons between the different parameters. The significance of comparisons between groups was determined using the Kruskal–Wallis followed by Mann–Whitney (for nonparametric data). The behavioral data were analyzed by analysis of variance (ANOVA) for sucrose preference and elevated plus maze for the effects of TBI x treatment x sex and with repeated measures 3 way-ANOVA for the effects of behavior, TBI x treatment for the RI test. Post hoc or planned pair comparisons were done where interactions were significant. The number of rats who came first to the feeder in the dominant–submissive behavior test and mortality rate was analyzed with a chi-square, Fisher’s exact test. Normally distributed data and continuous variables were presented as an mean ± SD. Nonparametric data were presented as a median ± inner quartile range. Results were considered statistically significant when *p* < 0.05.

## Results

### Mortality

The survival rate was registered in the first 30 days following intervention. The mortality rate in sham-operated control rats was 0% in both gender groups, which was significantly lower than male (10.71%, *p* < 0.05, *n* = 56, chi-square and Fisher’s exact test, 2-sided) and female (12.28%, *p* < 0.05, *n* = 57, chi-square and Fisher’s exact test, 2-sided) rats following TBI.

### NSS

There were no baseline neurological deficits observed in any of the rats before intervention. The sham-operated control groups did not show a statistically significant neurological deficit at any time point throughout the experiment. Compared to sham-operated controls, the NSS at 72 h after intervention was significantly greater in male (5.5(4-6.25), *n* = 30 vs. 0(0-0), *n* = 30, *U* = 16.5, *p* < 0.01, *r* = 0.88) and female (5(3.75-6), *n* = 30 vs. 0(0-0), *n* = 30, *U* = 1, *p* < 0.01, *r* = 0.91) rats after TBI, according to Mann–Whitney test. No statistically significant differences were found between male and female groups at 32 days after intervention, according to Kruskal–Wallis one-way analysis. The data are measured as a count and expressed as median and 25–75 percentile range.

### MRI-determined brain injury

At 72 h after intervention, rats that received TBI had significantly greater lesion volumes in females (4.46% ± 2.92% vs. 0.95% ± 2.63%, t(38) = 3.99, *p* < 0.01) and males (4.08% ± 3.06% vs. −0.3% ± 3.08%, t(38) = 4.52, *p* < 0.01), and volume of brain edema in the females (3.61%±2.36% vs. 0.41% ± 1.14%, t(38) = 5.45, *p* < 0.01) and males (3% ± 1.83% vs. −0.08% ± 1.89%, *t*(38) = 6.16, *p* < 0.01), compared to the sham-operated rats. Statistics were performed via a Student’s t-test and the data are expressed as a mean percentage or ratio of the contralateral hemisphere ± SD.

### Concentration of blood glutamate

**Fig. 2:**
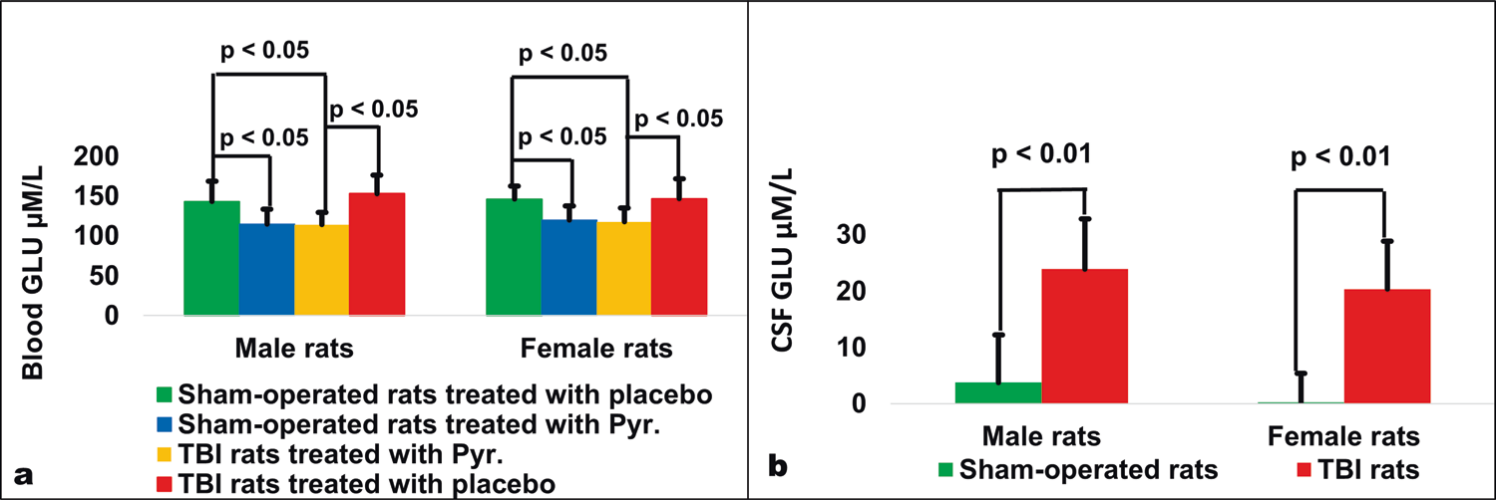
Blood and CSF glutamate levels. Blood glutamate in experimental group for males and females (**a**), and CSF glutamate levels following TBI compared to sham-operated controls in male and female (**b**) rats. For both male and female rats, there was a significant decrease in blood glutamate for sham-operated rats treated with pyruvate compared to sham-operated rats treated with placebo and post-TBI rats treated with pyruvate compared to post-TBI rats treated with placebo. Compared to sham-operated rats, the concentrations of CSF glutamate 12 h after surgical intervention was significantly greater in male and female rats following TBI. CSF Cerebrospinal fluid, GLU glutamate, Pyr Pyruvate, TBI Traumatic brain injury.

For male rats at day 17 after intervention, there were significant differences in blood glutamate levels between sham-operated rats treated with placebo (143.04 µM/L ± 25.79 µM/L), sham-operated rats treated with pyruvate (114.41 µM/L ± 19.12 µM/L), post-TBI rats treated with placebo (152.91 µM/L ± 23.55 µM/L) and post-TBI rats treated with pyruvate (113.81 µM/L ± 15.33 µM/L) according to a one-way ANOVA (F_3,36_ = 8.76, *p* < 0.01, *η*^2^ = 0.423). Post-hoc analysis with a Bonferroni test showed a significant decrease in blood glutamate for sham-operated rats treated with pyruvate compared to sham-operated rats treated with placebo (*p* < 0.05) and post-TBI rats treated with pyruvate compared to post-TBI rats treated with placebo (*p* < 0.01, Fig. 2a).

For female rats at day 17 after intervention, there were significant differences in blood glutamate levels between sham-operated rats treated with placebo (146 µM/L ± 16.57 µM/L), sham-operated rats treated with pyruvate (119.45 µM/L ± 18.07 µM/L), post-TBI rats treated with placebo (146.63 µM/L ± 25.17 µM/L) and post-TBI rats treated with pyruvate (117.52 µM/L ± 17.48 µM/L) according to a one-way ANOVA (F_3,36_ = 9.628, *p* < 0.01, *η*^2^ = 0.445). Post-hoc analysis with a Bonferroni test showed a significant decrease in blood glutamate for sham-operated rats treated with pyruvate compared to sham-operated rats treated with placebo (*p* < 0.05) and post-TBI rats treated with pyruvate compared to post-TBI rats treated with placebo (*p* < 0.05, Fig. 2a). The data are measured in µM/L presented as a mean ± SD.

### Concentration of CSF glutamate

Compared to sham-operated rats, the concentrations of CSF glutamate 12 h after surgical intervention was significantly greater in male rats after TBI (23.95 µM/L ± 8.87 µM/L vs. 3.74 µM/L ± 8.49 µM/L, t(38) = 7.37, *p* < 0.01, Fig. 2b) and female rats after TBI (20.36 µM/L ± 8.5 µM/L vs. 0.19 µM/L ± 5.19 µM/L, t(38) = 9.05, *p* < 0.01, Fig. 2b), according to Student’s t-test. The data are measured in µM/L and expressed as mean ± SD.

### Sucrose preference

**Fig. 3:**
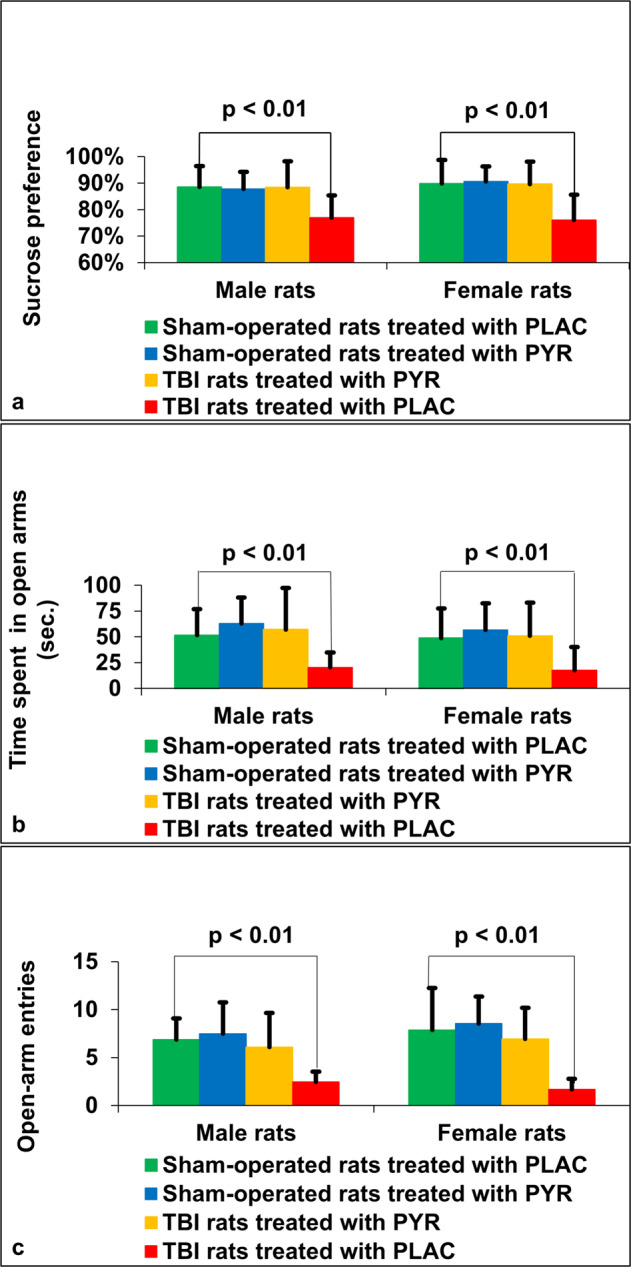
The sucrose preference and elevated plus maze results. **a** Sucrose preference. For male rats at day 32, sham-operated rats treated with pyruvate, sham-operated rats treated with placebo and post-TBI rats treated with pyruvate had significantly higher sucrose preference compared to post-TBI rats given placebo. For female rats, there was a significant increase between sham-operated rats treated with pyruvate, sham-operated rats treated with placebo and post-TBI rats treated with pyruvate compared to post-TBI rats given placebo. **b** Time spent on open arms. For male and female rats, there was a significant increase in the time spent on the open arms between sham-operated rats treated with pyruvate, sham-operated rats treated with placebo and post-TBI rats treated with pyruvate compared to post-TBI rats given placebo. **c** Open-arm entries. For male and female rats, there was a significant increase in the open arm entries between sham-operated rats treated with pyruvate, sham-operated rats treated with placebo and post-TBI rats treated with pyruvate compared to post-TBI rats given placebo. PLAC Placebo, PYR Pyruvate, TBI Traumatic brain injury.

The Levene’s test for homogeneity of variance for sucrose preference was not significant (*F* = 1.66, *p* = 0.33); therefore, the data were analyzed by a 3-way ANOVA for the effects of TBI (TBI or sham), treatment (pyruvate or control) and sex (male or female). ANOVA showed a significant main effect of TBI (F_1,112_ = 18.26, *p* < 0.01, _*p*_*η*^2^ = 0.14), a main effect of pyruvate treatment (F_1,112_ = 17.34, *p* < 0.01, _*p*_*η*^2^ = 0.13), and a significant interaction between TBI and pyruvate treatment (F_1,112_ = 17.21, *p* < 0.01, _*p*_*η*^2^ = 0.13). Planned comparisons confirmed that the TBI groups of both sexes from the control treatment had a lower sucrose preference compared to the sham-operated placebo group (F_1,112_ = 35.46, *p* < 0.000005). In contrast, rats that underwent TBI but were treated with pyruvate had a higher sucrose preference than the TBI rats given placebo (F_1,112_ = 34.55, *p* < 0.000005) and were not different from sham-operated rats (F_1,112_ = 0.008). No effect of sex or interaction between sex and TBI or pyruvate treatment were found (*F* < 1 in all cases). See Fig. 3a.

### Elevated plus maze

The Levene’s test for homogeneity of variance was not significant (*F* = 2.02, *p* > 0.05); therefore, the data were analyzed by a 3-way ANOVA for the effects of TBI (TBI or sham), treatment (pyruvate or control) and sex (male or female). ANOVA showed a significant main effect of TBI (F_1,112_ = 15.04, *p* < 0.01, _*p*_*η*^2^ = 0.12), a main effect of pyruvate treatment (F_1,112_ = 17.194, *p* < 0.01, _*p*_*η*^2^ = 0.13), and a significant interaction between TBI and pyruvate treatment (F_1,112_ = 7.60, *p* < 0.01, _*p*_*η*^2^ = 0.06). Planned comparisons confirmed that the TBI groups that received pyruvate spent more time in the open arms (54.05 ± 35.97, x̄ ± SD) than the TBI rats that were given the control (placebo) treatment (19.06 ± 18.49, x̄ ± SD). No effect of sex or interaction between sex and TBI or pyruvate treatment were found (*F* < 1 in all cases). See Fig. 3b.

The Levene’s test for the number of entries into the open arms was significant (*F* = 3.99, *p* < 0.01); therefore, the data were analyzed by the Kruskal–Wallis test. Since no sex difference was found in the time spent in the open arms, an initial Kruskal–Wallis test for the 6 treatment groups was done and confirmed that sex did not affect the number of open arm entries. Next, the effect of TBI and the effect of pyruvate was tested by grouping according to these factors, combining the males and females. There was a significant group difference (*F* = 55.01, *p* < 0.01). The TBI group treated with the control (placebo) had significantly fewer open arm entries than each of the other groups (TBI-pyruvate, sham-control, sham-pyruvate). The TBI-pyruvate group was not different from the two sham groups (vs sham pyruvate, *p* = 0.47; vs sham-control, *p* = 0.59; vs TBI-control, *p* = 0.01). See Fig. 3c.

### Resident-Intruder test

**Fig. 4:**
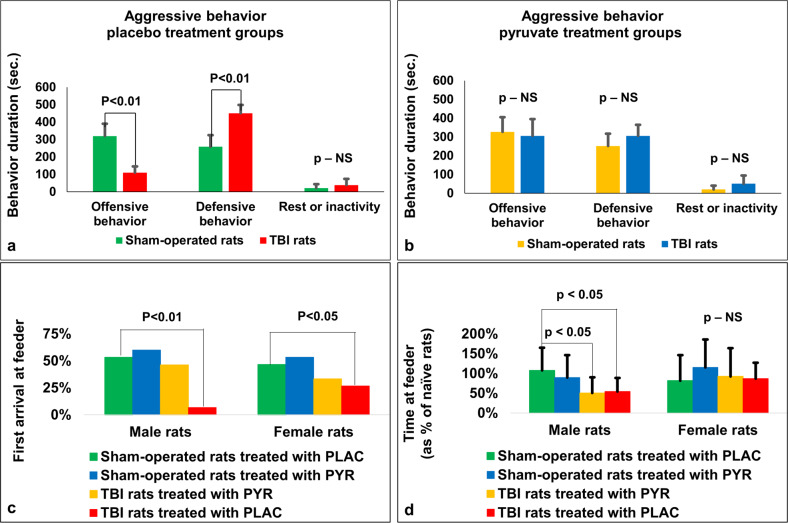
Results for the resident-intruder paradigm and assessment of dominant-submissive behavior. For the placebo group (**a**), TBI was shown to significantly decrease offensive behaviors and increase defensive behavior, with no effect on passive behavior. In rats treated with pyruvate (**b**), there was no significant change in offensive, defensive, or passive behaviors. There was a significant difference for female rats in the number of rats who came first to the feeder, for post-TBI rats given placebo (**c**). There was significantly less time spent at the feeder for male rats on the dominant-submissive task for post-TBI rats treated with pyruvate and post-TBI rats given placebo compared to sham-operated rats treated with placebo (**d**). There was no difference between groups in time spent at the feeder for female rats on the dominant-submissive task. NS not significant, PLAC Placebo, PYR Pyruvate, TBI Traumatic brain injury.

The 3 behaviors analyzed in the male rats exposed to the naïve “intruder” male, aggression, defensive behavior and passive behavior were analyzed by a repeated measures ANOVA for the effects of TBI vs sham and pyruvate vs control. There was a significant main effect of behavior (F_2,112_ = 254.50, *p* < 0.01, _*p*_*η*^2^ = 0.82) and a significant 3-way interaction between the effects of TBI, pyruvate, and behavior (F_2,112_ = 39.30, *p* < 000005, _*p*_*η*^2^ = 0.41). This interaction was further analyzed by planned comparisons, according to our hypothesis. First, the effect of TBI was shown to significantly decrease offensive behaviors (sham vs TBI control, F_1,56_ = 102.47, *p* < 0.01) and increase defensive behavior (sham vs TBI control, F_1,56_ = 76.95, *p* < 0.01), with no effect on passive behavior (F_1,56_ = 2.16, n.s., Fig. 4a). In order to test the effect of pyruvate on the TBI animals, we conducted a planned comparison between the TBI control and TBI pyruvate groups (Fig. 4b). Pyruvate increased offensive (F_1,56_ = 92.17, *p* < 0.01) and decreased defensive (F_1,56_ = 90.14, *p* < 0.01) behaviors in the TBI rats, compared to TBI treated with the control water. No effect of pyruvate was found for passive behaviors (F_1,56_ = 1.15, n.s.). Pyruvate had no significant effect on the sham operated rats for offensive (F_1,56_ = 0.08), defensive (F_1,56_ = 0.10, n.s), or passive behavior (F_1,56_ = 0.004). In summary, the effect of TBI to reduce offensive and increase defensive behaviors was reversed by pyruvate treatment, which had no effect on sham-operated rats.

### Assessment of dominant-submissive behavior

At day 41, Chi-square, Fisher’s exact test showed a significant difference in the number of rats who came first to the feeder only in female rats after TBI given placebo (4 from 15, *p* < 0.05, Fig. 4c) and male rats after TBI given placebo (1 from 15, *p* < 0.01, Fig. 4c). The data are measured in sec and presented as a percentage.

For male rats at day 41, a one-way ANOVA showed a significant difference in the time spent at feeder between the study groups (F_3,56_ = 4.663, *p* < 0.01, *η*^2^ = 0.209). Post-hoc analysis with a Bonferroni test showed significantly less time spent at the feeder between post-TBI rats treated with pyruvate (50.4% ± 39.6%, *p* < 0.05) and post-TBI rats given placebo (54.5% ± 34.1%, *p* < 0.05) compared to sham-operated rats treated with placebo (107.8% ± 57.4%, Fig. 4d). For female rats at day 41, in post-TBI rats given placebo (86.8% ± 40.1%), post-TBI rats treated with pyruvate (92.4% ± 71.6%), sham-operated rats treated with placebo (81.9% ± 64.4%) and sham-operated rats treated with pyruvate (115.5% ± 70.3%) a one-way ANOVA did not show a significant difference in the time spent at a feeder (F_3,56_ = 0.828, *p* = 0.484, *η*^2^ = 0.043, Fig. 4d). The data are measured in sec expressed as a percentage from naïve rats and presented as mean ± SD.

## Discussion

We explored the effects of the BGS pyruvate on the development of neuropsychiatric sequelae of rats with experimental TBI. We specifically studied anhedonia, anxious behavior, social impairment (including aggressive and hierarchical behavior) in rats that underwent TBI treated with pyruvate. To verify TBI, an MRI examination, determination of the concentration of glutamate in the CSF, and a neurological examination were performed. Treatment was monitored by analysis of blood glutamate concentration and the control of pyruvate consumption was made through drinking water. The main conclusion of this study is that pyruvate likely exerts brain neuroprotection via its BGS activity. For the first time, we show a preventative effect of post-TBI pyruvate treatment on the development of decreased aggressive and enhanced anxiety-like behavior and anhedonia in a rat model of traumatic brain injury. These effects were accompanied by evidence of pyruvate’s ability to remove glutamate from the blood.

As expected, rats from the TBI group had increased infarct volume, cerebral edema, levels of CSF glutamate, and mortality compared to the sham-operated group. In this population, we also detected neurological deficits at 72 h in both males and females that spontaneously resolved at 4 weeks post-injury. The blood glutamate concentration was monitored during the treatment period, confirming the reduction in blood glutamate levels due to pyruvate administration. TBI rats had post-TBI depressive-like symptoms, post-TBI anxious behaviors, and increased passive and submissive behavior compared to sham rats. These neuropsychiatric sequelae are a typical consequence of TBI and have been well documented in earlier publications in both humans [55–57] and rats [38, 58].

Prevention of the development of post-TBI depressive-like symptoms with pyruvate replicates our previous finding that pyruvate prevented depressive behaviors post-TBI [38] as well as post-stroke [22] in a rat model. BGS has been proposed as a new and promising treatment for patients with depression [18].

The results of the plus maze test (time in the open arms and number of entries into the open arms) showed that TBI rats treated with pyruvate did not develop post-TBI anxiety in contrast to TBI rats receiving placebo. Sham rats receiving pyruvate did not differ from sham rats receiving placebo, confirming that the effect of pyruvate is specific to anxiety induced by TBI and not as a result of a general increase in activity or impulsivity [38]. Both males and females responded positively to pyruvate treatment and showed similar results. Thus, in this experiment, the administration of pyruvate clearly showed an anti-anxiety effect in a rat model of post-TBI anxiety.

SSRIs and SNRIs are known to be appropriate treatments for depression and are also recommended as first-line treatments for anxiety. Our results suggest that the administration of pyruvate, which has glutamate-based antidepressant effects, can have similar efficacy for treating anxiety [59]. These types of treatments are particularly helpful for conditions in which anxiety occurs by prolonged dysregulation of brain glutamate due to combined BBB disruption and neurotoxicity, which happens in TBI, stroke and similar conditions [60].

Interferences in social behavior, including aggression, apathy, impaired social communication, difficulties in social relationships, personality changes, and hallucinations are often associated with the consequences of TBI in humans [57]. For the assessment of social impairments in a TBI rat model, we used the resident-intruder test and the assessment of hierarchical relationships, tests which provide excellent predictive validity for human aggression [61].

The test results demonstrated a reduced level of offensive behavior in TBI rats compared to sham rats, which was alleviated by pyruvate treatment. The pyruvate-treated TBI group did not show less offensive behavior than the sham group. In addition, we documented an increased level of defensive behavior in rats exposed to TBI that were treated with placebo, but not in TBI rats treated with pyruvate. Defensive behavior in TBI rats that received pyruvate did not differ from defensive behavior of sham rats. The resident-intruder test is used in the male population, so we did not test females for this test.

Apathy, aggression, and other disorders of social behavior can be both a primary diagnosis and a consequence of depression and anxiety. Aggressive behavior in depressive-anxious patients is often described as the result of a reaction to an irritant and would be more represented in a paradigm of defensive or aggressive behavior, and not as a result of initially initiated attacks. A similar paradigm of behavior is present in the animal population [61, 62], and the involvement of glutamate in aggression has also been highlighted in the scientific literature [63, 64]. Drugs that act on the glutamate system have been shown to be effective in reducing aggressive behavior in mice [63]. In our experiment, we cannot say with certainty whether the changes in the social behavior of rats were a direct consequence of TBI, or whether it was a consequence of the depressive-anxious state of the rats, or the combined effect of these two factors. In any case, TBI rats responded effectively to pyruvate treatment and did not differ statistically from sham rats in both defensive and offensive behavior.

The results of the evaluation of the “first arrival at feeder” showed that TBI rats administered pyruvate did not develop submissive behavior, in contrast to TBI rats treated with placebo and compared with naïve ones. Pyruvate-treated sham rats did not differ from placebo-treated sham rats or naïve rats. Both males and females in the TBI group responded positively to pyruvate treatment and showed similar results. Therefore, we can conclude that treatment with pyruvate prevented the change in hierarchical behavior typically associated with TBI. The results of the “time at feeder” test is less clear, however. Placebo-treated male TBI rats spent less time on the feeder compared to sham rats. But the females showed no significant difference between the experimental groups. A possible explanation is that the manifestation of hierarchy may be different for males and females, so a different set of tests and assessment methods should be applied. For example, protection and care for offspring is an indicative test for female rats, but is not applicable for male rats [65], and territorial hierarchy is a test for males, but not for females [66, 67].

A limitation to this study was that we did not study inflammation and neurodegeneration, which play an important role in the development of depression, anxiety, and other neuropsychiatric complications of TBI [68]. The pathogenesis of post-TBI neuropsychiatric disorders is complex and multifactorial [16]. Future studies should further examine the multimodal pathogenesis of post-TBI psychiatric consequences, including the role of inflammation in the context of our findings.

In conclusion, we believe that drugs focused on lowering brain glutamate levels may be effective in treating anxiety and depression, and modulating TBI-related changes in social behavior. The use of BGS for the treatment or prevention of psychiatric illness would have particular relevance in cases of BBB dysregulation, such as head trauma, stroke, and similar conditions.

## Data Availability

The data that support the findings of this study are available from the corresponding author, [MB], upon reasonable request.
